# Is There Any Evidence for Rapid, Genetically-Based, Climatic Niche Expansion in the Invasive Common Ragweed?

**DOI:** 10.1371/journal.pone.0152867

**Published:** 2016-04-26

**Authors:** Laure Gallien, Wilfried Thuiller, Noémie Fort, Marti Boleda, Florian J. Alberto, Delphine Rioux, Juliette Lainé, Sébastien Lavergne

**Affiliations:** 1 Laboratoire d’Écologie Alpine (LECA), Univ. Grenoble Alpes, F-38000, Grenoble, France; 2 Laboratoire d’Écologie Alpine (LECA), CNRS, F-38000, Grenoble, France; 3 Conservatoire Botanique National Alpin, Domaine de Charance, 05000, Gap, France; University of York, UNITED KINGDOM

## Abstract

Climatic niche shifts have been documented in a number of invasive species by comparing the native and adventive climatic ranges in which they occur. However, these shifts likely represent changes in the realized climatic niches of invasive species, and may not necessarily be driven by genetic changes in climatic affinities. Until now the role of rapid niche evolution in the spread of invasive species remains a challenging issue with conflicting results. Here, we document a likely genetically-based climatic niche expansion of an annual plant invader, the common ragweed (*Ambrosia artemisiifolia* L.), a highly allergenic invasive species causing substantial public health issues. To do so, we looked for recent evolutionary change at the upward migration front of its adventive range in the French Alps. Based on species climatic niche models estimated at both global and regional scales we stratified our sampling design to adequately capture the species niche, and localized populations suspected of niche expansion. Using a combination of species niche modeling, landscape genetics models and common garden measurements, we then related the species genetic structure and its phenotypic architecture across the climatic niche. Our results strongly suggest that the common ragweed is rapidly adapting to local climatic conditions at its invasion front and that it currently expands its niche toward colder and formerly unsuitable climates in the French Alps (i.e. in sites where niche models would not predict its occurrence). Such results, showing that species climatic niches can evolve on very short time scales, have important implications for predictive models of biological invasions that do not account for evolutionary processes.

## Introduction

Biological invasions alter the structure of native communities and can disturb ecosystem functioning worldwide [1]. The ever-increasing spread of invasive species has thus stimulated an important body of research [2,3]. It notably becomes of increasing interest to understand how evolution shapes species' climatic niches, as it will shed light on the determinants of invasions under current and future climates. This is particularly important for developing forecasting tools for biological invasions [4,5]. Indeed, it remains unclear whether, over short time scales, the niche of invasive species can be considered as a fixed characteristic or whether they can experience rapid evolution, as shown for other species characteristics (e.g., [6] for life history traits; [7] for several niche related traits).

Shifts in climatic niches have already been documented in a number of invasive species, by comparing the native and adventive climatic ranges in which they occur [8,9]. However, these shifts likely represent changes in the realized climatic niches of invasive species, and may not necessarily be driven by genetic changes in climatic affinities [10]. Nonetheless, other studies have shown that some introduced plant species have quickly adapted along climatic gradients [3], but the evidence that invasive plant species can adapt to novel climates is still lacking–that is, niche expansion. Such adaptive processes being shaped by the interplay of introduction history, genetic makeup of introduced populations, gene flow and niche-related trait variation.

The critical processes allowing niche and range expansion are known to occur at species' range margins [11]. On the one hand, repeated introductions and important propagule pressure may help overcome demographic bottlenecks, and ultimately enhance adaptation in introduced organisms [12]. This can also generate novel genotypes through recombination and increase evolutionary potential at adaptive loci [13], or simply increase the fitness in invasive populations through hybrid vigor [14]. On the other hand, high gene flow between invasive populations may provoke gene swamping in climatically marginal populations and prevent them from developing local adaptation [15]. Furthermore, the evolvability of invasive populations towards novel climatic niche space depends on the level of genetic variation in climatically relevant functional traits, but also on their genetic covariances relative to the main directions of the newly experienced selective pressures. Indeed, when several traits are considered together, the number of combinations that can respond to selection can be much smaller than the number of traits, limiting or preventing the evolution of an optimal combination of traits [16]. To really identify when new genetic makeup of invasive populations affect climate-related functional traits, and whether phenotypic changes observed at the forefront of invasive species range are of any adaptive significance, data on geographic distribution, phenotypic traits and molecular markers can then be used all together to disentangle adaptive from non-adaptive trait changes across a niche gradient (e.g., [17,18]). Indeed, populations adapting to new environmental conditions should show both: genetic signatures independent of genes dispersal limitations, and genetically based phenotypic variations.

**Fig 1:**
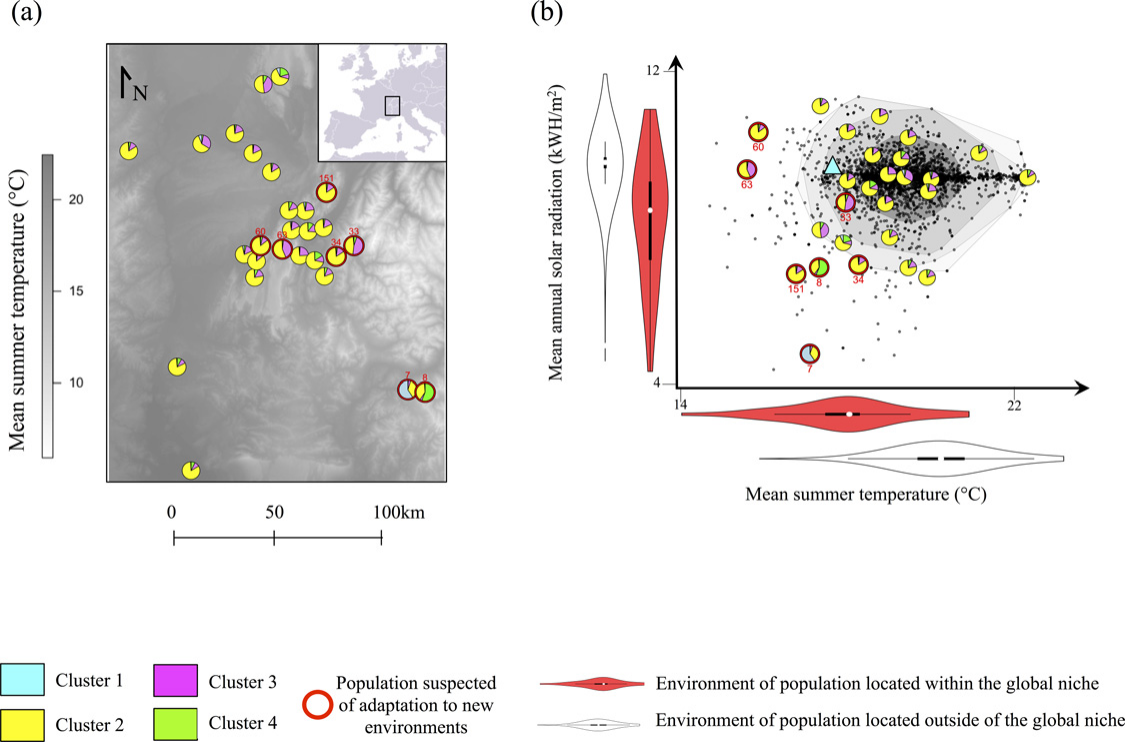
Location of the 27 populations sampled for the genetic analysis. Populations are presented in (a) the geographic space, plotted over a map of the mean summer temperature and (b) the regional niche space. Each population is represented with a pie chart showing the average proportion of the genetic clusters inferred from structure in each population. The populations *a priori* suspected of adaptation to novel climates are circled in red. For (b) the dark grey dots indicate the position of the 3,888 populations of *A*. *artemisiifolia* recorded in the French Alps, used to estimate the regional niche of the species. A blue triangle represents the location of the common garden experiment.

Here, we developed an original approach combining species’ climatic niche models at different spatial scales, population genetics and a common garden experiment to identify and localize rapid climatic niche expansion in the common ragweed (*Ambrosia artemisiifolia* L., Asteraceae) at its upward migration front in the French Alps. We first measured both the climatic niche of the invader in the French Alps (*regional niche*), and its global climatic niche that combines all known native (North America) and introduced ranges throughout the world (*global niche*). This global niche estimates the full range of environmental conditions under which the species has been observed to survive, and thus approximate the “true” physiological limitations of the species, that is its fundamental niche [4]. Combining these two niche estimates allowed us identifying the populations that can be suspected of contemporary climatic niche expansion in the French Alps (i.e. the populations occurring within the regional niche but outside of the global niche, as shown in [10]; Fig 1, S1 Appendix). The regional niche estimation was used to stratify the sampling of genetic data and seed material [19] notably in identifying populations at the most extreme climatic niche edges. Second, we used genetic markers to infer both the neutral genetic structure due to demographic processes, and potential genomic signatures of natural selection. Third, based on seeds collected across the whole regional niche, we measured phenotypic traits in a common garden to test whether genetic variation in niche-related functional traits related to genomic signatures of selection, and whether phenotypic architecture (i.e. traits variances and covariances) might facilitate or impede adaptive evolution towards colder, formerly unsuitable, climatic conditions.

## Materials & Methods

### The studied species

The common ragweed (*Ambrosia artemisiifolia* L., Asteraceae) is a North American diploid annual weed that was transported with seed crops and forages about 150 years ago into several distinct locations across Eurasia, Australia and South America. The species has been continuously spreading since then, reaching the French Alps about sixty years ago [20]. It is a highly colonizing species, reproducing through wind-pollination with a self-incompatible breeding system, and long seed dormancy [21,22]. The species is essentially dispersed by human activities and tend to mainly occur in frequently disturbed habitats such as roadsides and agricultural fields [23]. Interestingly, this species usually occurs at low elevations with mild climatic conditions in its native range but has been recorded at increasingly elevated locations over the past decade in the French Alps (national botanical survey, CBNA). This suggests on-going adaptation to colder conditions not encountered by the species elsewhere in the world [10].

### Inferring climatic niches at different geographic scales

The climatic niche of the study species was estimated at both global (*i*.*e*. including the native and all invaded ranges; Fig B in S1 Appendix) and regional scales (*i*.*e*. French Alps), according to a former work [10]. The global niche estimation procedure was based on an ensemble forecast [24] using 4,803 occurrence of *A*. *artemisiifolia* over the world. These distribution data were assembled from the Global Biodiversity Information Facility (GBIF, http://data.gbif.org) at a minimum resolution of 2.5 arc-minutes (c. 4.5 km), and 20,000 background data from realistically reachable locations (within a buffer zone of 20 km around any presence record). Five uncorrelated climatic variables were extracted from WorldClim at a resolution of 2.5 arc-minute (http://www.worldclim.org [25]): maximal temperature of the warmest month, annual temperature range, mean temperature of the coldest quarter, sum of precipitations of the wettest month, and sum of precipitations of the driest month. These variables were chosen because the occurrence of common ragweed is generally limited by strong summer drought (i.e. high temperature and low precipitations), short vegetation periods, and high soil moisture. We refer to Gallien *et al*. [10] for further details on the global niche estimation.

The regional niche for the French Alps was built using 3,888 observations of *A*. *artemisiifolia* (source CBNA) at 100m resolutions and 10,000 background data randomly selected in the study region, with the same procedure as for the global niche. Because we wanted to do both: stratify our sampling with the regional niche model, and provide an ecological understanding of the evolutionary processes occurring along the regional niche gradients, we needed to use a restricted number of environmental variables to estimate this niche. Based on a principal component analysis (PCA) run over 21 climatic and topographic variables at a resolution of 100m (see S1 Appendix for more details on the variables), we selected the two variables that most segregated the sites occupied by the species in the French Alps: mean annual solar radiation (a measure highly related to the aspect of the sites) and mean summer temperature (i.e. the mean monthly temperatures over the three summer months: June, July, August). Both variables were highly correlated with first two axes of the PCA and explained 70% of the inter-sites differences. Based on the same ensemble modelling as for the global niche estimation we confirmed that this regional model was well performing (AUC = 0.94; [26]).

The global *vs*. regional niche comparison was then used to identify populations suspected of adaptation to novel climatic conditions experienced nowhere else by the study species (Table B in S1 Appendix; [10]). Indeed, populations that occur in environmental condition predicted to be outside of the global niche of the species could either be demographic sinks or recently adapted populations. However, since they are well predicted by the regional niche model suggests that they are not sink populations. It can be noted that, for building the global niche model we used populations from the native range as well as from other invaded regions. Therefore the global dataset may contain both: populations that are adapting to new local climatic conditions in invaded ranges (though unlikely as shown by [27]), and sink populations. However, the addition of such populations into the global niche estimate should just enlarge the global niche edges, and make the detection of locally adapting populations in the region of interest less likely. Hence we believe that our global niche measure is more over-estimated than under-estimated, and thus our comparison of global and regional niches is conservative (see S1 Appendix for more details on this comparison).

### Genetic structure across the niche

During summer 2010 we collected leaf samples within 27 populations (10 individuals per population) selected to be representative of the regional niche, including seven ones which were predicted to occur out of the species global niche (no specific permission were required for sampling at these locations since the studied species is a noxious invasive weed). The sampling was designed in a way that populations in similar environmental conditions were not spatially clustered (Fig 1). After DNA extraction and allele scoring, 240 AFLP markers were kept for analyses (S1 Appendix). First, we tested whether some markers exhibited putative selection signatures related to the two main regional niche gradients using logistic regressions (controlling for spatial autocorrelation and multiple testing, see S2 Appendix). It can be noted here that we did not use bayescan to identify putative markers under selection since it does not perform well for gradual selection along environmental gradients [28]. Second, after removing the markers potentially under selection, we estimated three population-specific genetic parameters with bayescan: inbreeding coefficient F_IS_, genetic differentiation F_ST_, and genetic diversity H_e_ [29] (see S1 Appendix for more details on the method). The relationship between F_IS_, F_ST_ and H_e_ and the main two regional niche gradients (temperature and radiation) were then tested using regression models (allowing for linear and/or quadratic relationships) following a stepwise AIC procedure. Since genetic diversity should decrease as a result of selective pressures, we expected that H_e_ would be lowest at sites where the species is suspected to be adapting to new environmental conditions, and away from source populations. Similarly, F_ST_ should increases in populations highly differentiated from their ancestral populations (i.e. source populations), we thus expected a high F_ST_ in populations at the leading invasion front if they are little connected by dispersal. To investigate the genetic structure of the populations we used the Bayesian clustering program structure 2.3 [30] from which we identified the optimal number of clusters (S1 Appendix). Third, to identify the barriers to gene flow between populations, we also tested for genetic isolation by geographic (IBD_geo_) and environmental distances (IBD_env_, where environmental distance is estimated as the Euclidian distance between population along the two regional niche axes) using Mantel tests with 999 randomizations (R package *ade4* [31]).

### Phenotypic structure across the niche

Among the 27 populations used for the genetic analyses, we chose 18 that were evenly distributed across the regional climatic niche space, including 4 populations that were suspected of climatic niche expansion. In each population, 30 seeds were collected from each of the 6 randomly chosen mother plants during fall 2010. Based on these seeds, we conducted a common garden experiment (detailed protocol in S1 Appendix), located in Gap (French Alps; see Fig 1 for its position in climatic space) This experiment was based on about 3000 plants randomly placed into 3 rows of 10 blocks containing each about 100 individuals. Given the large size of the experiment, only half of the plants were randomly chosen to take morphological measurements (ca. 1134 measured plants). Plant height was measured every two weeks for the entire duration of the experiment (for further indirect test of maternal effects, S2 Appendix). Right before the flowering period (2.5 month growth, to avoid pollen spread), plants were collected to measure four quantitative traits: total dry biomass, plant height, shoot-root dry biomass ratio, and leaf dry matter content (LDMC, leaf dry mass/fresh mass; S1 Appendix). These four traits were chosen to reflect local adaptation toward more stressful conditions, such as cold temperatures and low levels of solar radiation. In such conditions, plants are expected to show greater allocation towards root tissues (notably because of reduced rates of water and nutrient uptake; [32,33]), lower biomass production (notably because of shorter growing seasons and lower resource availability; [34]), and increased leaf dry matter content (which increases leaf life span and resource economy; [35]). It is important to keep in mind that due to its highly allergenic pollen, measuring more direct fitness related traits as well as obtaining a second generation of *A*. *artemisiifolia* in common garden or greenhouse conditions was not allowed by regional regulations: strong health issues for experimental workers and surroundings had to be anticipated. Therefore, we could also not control for maternal and epigenetic effects (but see S2 Appendix for indirect tests of maternal effects).

Assuming that interference from maternal and epigenetic effects on phenotypic variation was negligible (see Discussion), we first estimated the overall genetic differentiation among populations Q_ST_ (see S2 Appendix for details on the calculations) for each trait. We then compared each Q_ST_ value to the F_ST_ value calculated from neutral markers in order to identify the traits putatively under selection. Second, we tested whether the traits changed over the regional niche of the species using linear mixed models. Both temperature and radiation were included in simple and quadratic forms as fixed effects as well as their interactions, along with population, family and block-design as random effects. The models were then tested within a stepwise AIC procedure (S2 Appendix). Third, to estimate whether genetically based phenotypic correlations limit the ability of some populations to respond to selection, we estimated the additive genetic variance-covariance matrix ***G*** of each population [36] using MANOVAs (code modified from [37]). From ***G***, we identified three major descriptors of genetic variance: (1) the *total genetic variance*, or ***G***'s volume, measured as the sum of all trait variance, (2) the strength of *genetic correlation*, or ***G***'s shape, taken as the proportion of the total genetic variance explained by the largest eigenvector *Pmax*, and (3) the *direction of the genetic correlations* ('line of least resistance' [38]) estimated as the direction of *Pmax* vector. For the first two indices, we tested if they varied along the niche gradients, using generalized linear models. For the direction of *Pmax*, we tested whether populations from similar climate presented similar *Pmax* directions, using a correlogram (S2 Appendix). Additionally, to assess the sensitivity of Q_ST_ and ***G*** matrix estimates to the low number of sampled seed families we applied a jack-knife procedure over the seeds families (Table C and Fig G in S2 Appendix).

### Potential response to selection

Assuming that most variation in ***G*** matrices captures genetically-based phenotypic variation (see discussion), we finally applied the Selection Skewers Method (SSM) [39] to estimate how the potential of phenotypic evolution changes towards niche edges. SSM uses a multivariate breeder's equation to estimate the response to selection of a ***G*** matrix (more details in S2 Appendix). Our goal here was to test whether populations at the niche edge can pursue adaptation toward more stressful conditions, while accounting for trait variance and covariances. We applied one selection scenario to estimate the population responses to selection toward colder and lower levels of solar radiation conditions, using a vector of selection computed as the estimated coefficient of regression between the niche gradients and each standardized phenotypic traits. More specifically, the response to selection is specified by the multivariate equivalent of the breeder's equation: *z* = ***G***β where *z* is the vector of mean trait response to selection and β is the vector of selection gradients.

### Linking traits with signatures of selection

To estimate whether the functional traits could be linked with some of the markers putatively under selection we calculated the Spearman rank correlation coefficient between each predicted trait value per population (using the trait-environment regressions) and the predicted allelic frequency in the same populations (using the allele-environment regressions). It is important to note that observed associations between allele frequencies and traits are unlikely to be biased by the neutral genetic structure of populations because this structure is almost inexistent (see Results section) and because our sampling design aimed at decoupling environmental from spatial effects.

## Results

### Spatial patterns of neutral and selected genetic variation

A total of 240 AFLP markers were scored in the 27 populations scattered across the species regional range and climatic niche. Among these markers, 36 (15%) were considered as potentially under selection, as showing a significant relationship (linear or quadratic) with at least one of the two main niche gradients of *A*. *artemisiifolia*: temperature and radiation. Using a conservative approach, all 36 markers were excluded from the analysis of the neutral genetic structure (since these analyses usually assume that the markers used are not under selection). Analyses of allele-climate and allele-trait associations (see below) were performed for a subsample of 29 markers (out of 36) that satisfied a false discovery rate below 5% (S2 Appendix).

**Fig 2:**
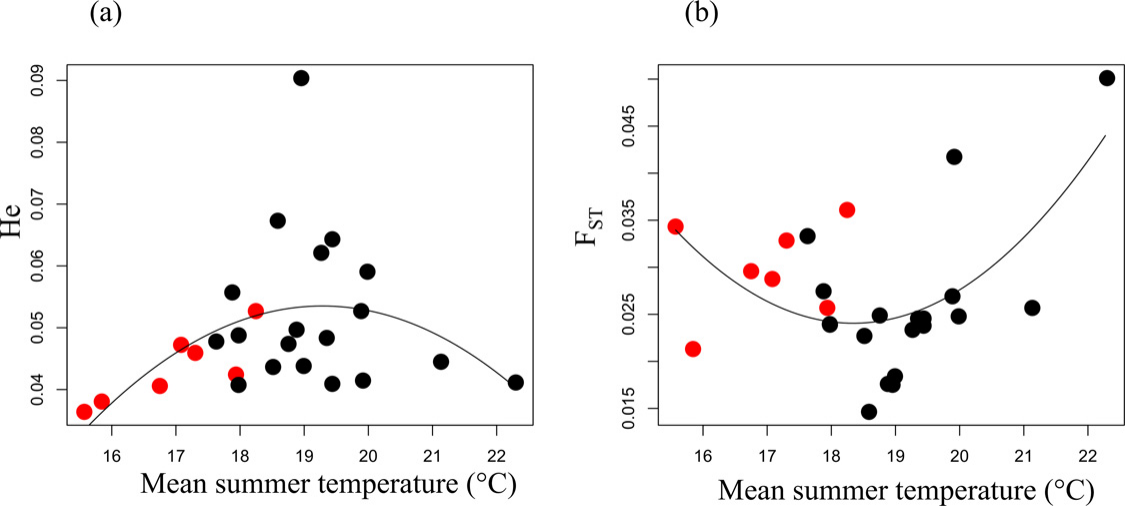
Population genetic characteristics along environmental gradients. Neutral genetic diversity H_e_ within populations (a) and population-specific genetic differentiation F_ST_ (b) as a function of the mean summer temperature experienced by each sampled populations. The populations *a priori* suspected of adaptation to novel climates are plotted in red.

Based on the remaining 204 neutral markers, we obtained generally high inbreeding coefficients within-populations (F_IS_ = 0.441 on average, ranging from 0.110 to 0.700), but they were not significantly correlated to the niche gradients. Intra-population genetic diversity H_e_ varied from 0.035 to 0.090, and showed maximal values at the core of the temperature gradient while being reduced at the cold and warm edges (Fig 2A). Estimates of population differentiation F_ST_ ranged from 0.015 to 0.050, and tended to be higher at the edges of the niche (p-val = 0.030; Fig 2B). The general level of genetic differentiation among the 27 populations was low but significant (F_ST_ = 0.021; p-val<0.001), suggesting extensive gene flow between populations.

Still on the 204 neutral markers, four clusters were detected by applying the clustering algorithm STRUCTURE, with high but homogeneous levels of admixture within each population (Fig D in S2 Appendix). Globally, individuals in most of the populations were assigned mainly to cluster 2 and then to cluster 3 (respectively 72.2% and 17.2% on average), except for two populations showing admixture with cluster 1 and 4 (see S2 Appendix). More details on individual assignments can be found in S1 Appendix. The individual assignment probabilities to the four clusters were not significantly correlated with the two main niche gradients. No significant isolation by environmental or geographic distance was detected in our pool of neutral markers (IBD_env_ p-val = 0.893 and IBD_geo_ p-val = 0.787), suggesting that little genetic structure exist due to spatial or environmental isolation. On the contrary and as expected, the markers suspected of adaptation (or linked to locus involved in adaptation) showed significant IBD_env_ (p-val = 0.008) and non-significant IBD_geo_ (p-val = 0.259).

### Phenotypic architecture across the niche

**Fig 3:**
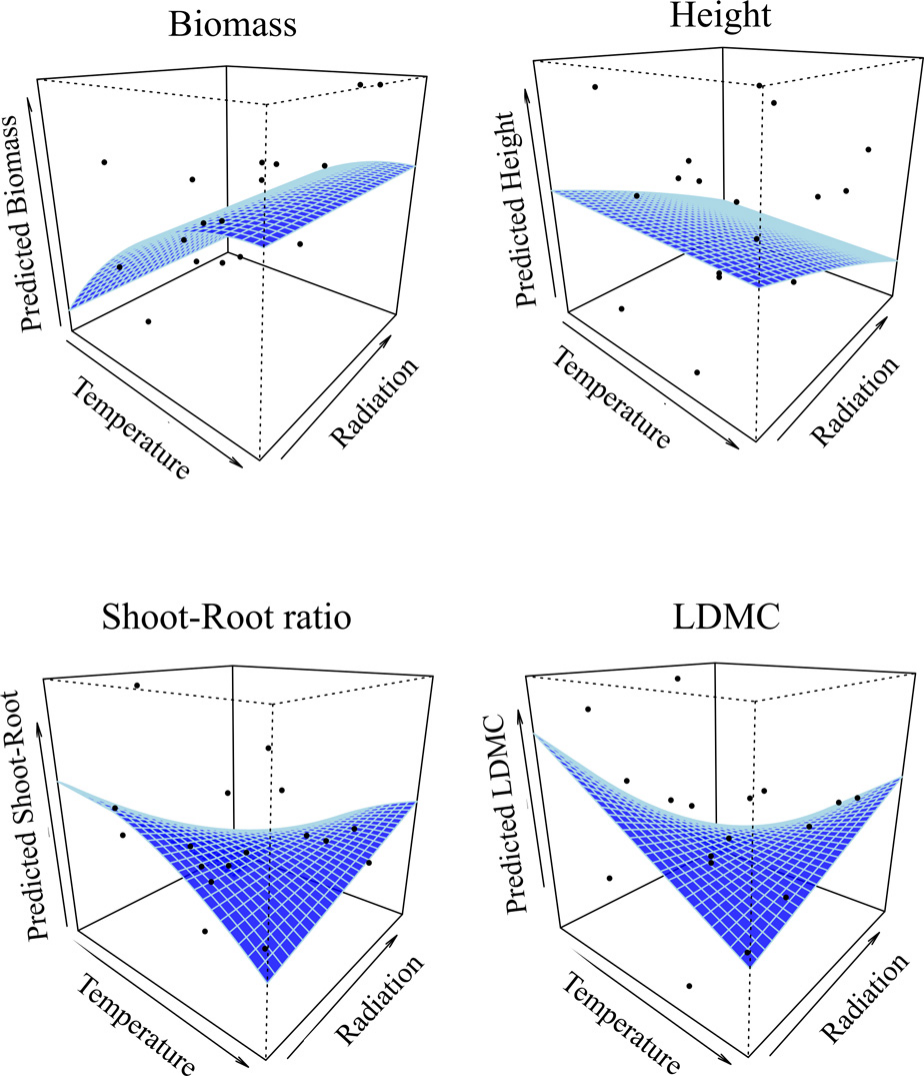
Relationship between each of the four functional traits measured in common garden and the two axes of regional niche gradients. The two environmental gradients are mean summer temperature and mean annual solar radiation. Curves were estimated from generalized mixed effect models taking into account the population structure and the experimental design random effects. The black dots represent the mean trait value for each population.

Experimental blocks within our common garden had significant effects on the measured traits and were thus included in all further analyses (Table B in S2 Appendix). We also found little potential influence of maternal effects on traits measured in common garden (S2 Appendix). Assuming a uniform half-sib genetic structure within all seed families, the estimated levels of genetic differentiation for phenotypic traits were rather low for biomass, height, shoot/root ratio (Q_ST_ = 0.074, 0.078 and 0.069, respectively; Table C in S2 Appendix) but much higher for leaf dry matter content (LDMC, Q_ST_ = 0.207), an important trait for adaptation to abiotic stress [40]. The genetic differentiation of all phenotypic traits was found to be higher than the average one inferred from neutral markers (i.e. Q_ST_ > F_ST_), suggesting divergent selection on functional traits at the regional scale, potentially due to climatic gradients. Biomass was indeed related to the temperature gradient with an asymptotic relationship (reaching a maximum at high temperatures), while plant height was nearly not affected by temperature but negatively correlated with radiation. Plant shoot-root ratio and LDMC were both negatively correlated with temperature but positively with radiation (Fig 3; see Table D in S2 Appendix for estimated parameters).

**Fig 4:**
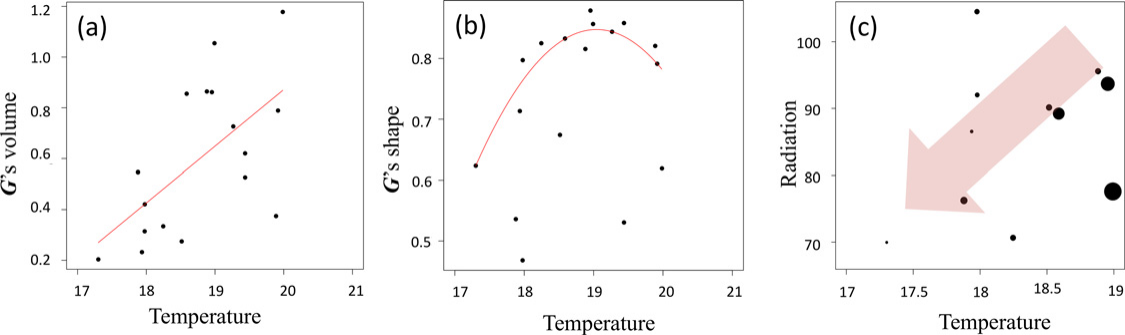
Phenotypic variance and integration across the species' niche as captured by the temperature gradient. The three panels represent different features of the so-called phenotypic ***G*** matrix (i.e. the traits genetic variances and co-variances). (a) The relation between ***G****'s* volume (i.e. total genetic variance) per population and the temperature gradient. (b) The relationship between ***G****'s* shape (proportion of variance explained by *Pmax)* and the temperature gradient. (c) The relationship between the population potential response to selection toward colder and low levels of solar radiation, and two regional niche axes: temperature (in °C) and solar radiation (in kWH/m^2^; only considering population of the coldest half of the gradient). The size of the dots represents the mean absolute trait displacement after application of the Selection Skewer Method, and the red arrow indicates the direction of the applied selection vector.

We then explored patterns of quantitative genetic variation by examining how the size and structure of the ***G*** matrix evolved along niche gradients. We found that the total genetic variance (i.e. ***G’***s volume) significantly decreased with decreasing temperature (R^2^ = 0.41; Fig 4A), indicating that population's evolvability (at least for this combination of traits) tended to decrease during its colonization towards colder environments. Then we detected that *P*_*max*_ was strongly linked with biomass and that the percentage of variance explained by *P*_*max*_ (i.e. ***G***'s shape, that is phenotypic integration) was lower for cold conditions (R^2^ = 0.24, Fig 4B). That is to say, trait correlations were weaker in colder environments. We also observed that the direction of the phenotypic integration *P*_*max*_ varied according to the temperature gradient, and that the populations of particularly cold conditions showed convergent phenotypic integration, probably due to similar strong directional selection (Fig F in S2 Appendix). Lastly, selection skewer analyses showed that when the direction of the selection pressure was directed toward colder and lower levels of solar radiation, populations located at the cold niche edge have a reduced potential for adaptive response than populations sampled at species' niche core (Fig 4C). These results suggest that past selective pressures have depleted the species adaptive potential at the upward migration front.

**Fig 5:**
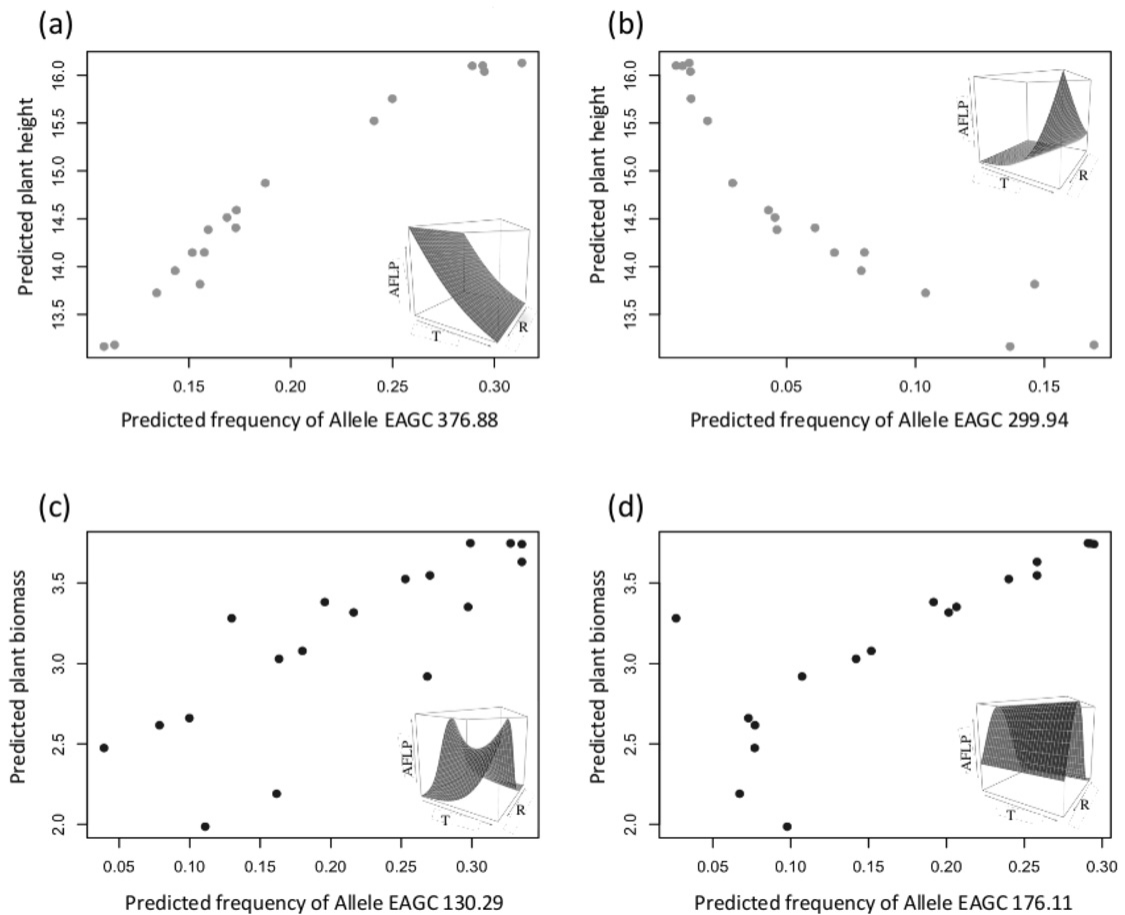
Four examples of strong correlations between estimated population AFLP marker frequency and estimated population functional trait value (correlation coefficient > 0.8). The first line (a-b) shows two correlations between the population predicted allelic frequency with the predicted plant height, and the second line (c-d) shows two correlations with the predicted plant biomass. In the upper right corner of the four graphics is represented the allele environment relationship (T: temperature, R: radiation).

Finally, we sought for associations between phenotypic traits and the 29 markers that were potentially under selection. We found that allele frequencies of 8 markers were highly correlated with quantitative trait values (correlation coefficient > 0.8; Fig 5): mainly with plant height (5 markers) and plant biomass (3 markers). These 8 markers are thus likely linked with genomic regions under environmental selection, and would be of further interest for studying the genomic bases of *A*. *artemisiifolia* adaptation along the niche gradients.

## Discussion

Over the last decade, several studies have demonstrated that rapid adaptation can occur on short time-scales and fuel up the range expansion of invasive species into new regions [2]. In plants, main evidences have so far concerned adaptive changes either in response to new biotic conditions (e.g., lack of natural enemies [41]), or of life-history traits favoring colonization potential [13]. Here, our results suggest that invasive plants could also evolve towards novel climatic environments and thus expand their climatic niche. Even if some of our results should be taken with caution due to potential limitations (discussed below), our study has important implications for understanding whether and how rapid niche evolution can foster species invasions along climatic gradients [42] and thus amplify their adverse effects on native biodiversity [43].

### Pervasive local climatic adaptation despite extensive gene flow

We built our sampling in a highly heterogeneous alpine region, along steep climatic gradients and different valleys separated by high mountain ranges. Our data show important levels of genetic admixture within populations and limited neutral genetic structure across space, as exampled by the absence of genetic isolation by geographic distances. It corroborates previous findings that common ragweed was repeatedly introduced and experienced pervasive post-introduction admixture [44,45]. Our results indicate that gene flow homogenized genetic diversity among most populations, despite apparent physical barriers to dispersal. This lack of spatial isolation can emerge either from recurrent gene flow between established populations probably through passive human transportation, or alternatively through recurrent dispersal events of particular areas as a large diffusive population. Previous evidence, as well as our own results, better support a scenario of subsequent and time-discrete colonization events with relative high gene flow between populations [44,45]. Such a gene flow between populations is generally expected to act against local adaptation, especially when marginal (e.g., front) populations are small [11].

Despite extensive homogenizing gene flow, we found several lines of evidence for molecular and phenotypic signatures of adaptation along the temperature gradient. The rather high proportion of molecular signatures observed (12% after controlling for false discovery rate) could be explained by the *a priori* identification of the climatic gradients shaping the species' niche, which was used to design the sampling strategy. In our study, the potentially confounding effects of allele surfing, which can generate spurious allele-climate relationships [46], are quite unlikely as we purposely sampled climatically similar populations apart across geographic space. Thus, although genome scans are not a definitive evidence of local adaptation, the finding of several outlier loci despite extensive homogenizing gene flows between populations suggest that these experience drastic selective pressures in mountain environments.

We found higher phenotypic differentiation between populations than expected from demographic processes, with clinal trait variations across temperature and radiation gradients. Assuming that our common garden experiment adequately captured genetic variance, these relationships were conform to general expectations for adaptations to environmentally stressful conditions. Biomass and individual height reduction may enable plants to resist to low temperatures and high radiation and to complete their life cycle during shorter growing seasons (i.e. at high elevations). A greater investment in the above ground biomass may permit a stronger allocation to reproduction, and high leaf dry matter content confer resistance to stress by efficiently limit tissue degradation. Overall, this suggests that directional selection has acted on these traits across the species' climatic niche, favoring phenotypes with smaller stature and longer-lived leaves in populations of migration front.

Directional selection may have been particularly important in the populations located out of the species' global climatic niche (cold and low solar radiation conditions) where estimated phenotypic evolvability has been reduced by more than 30%, relative to core and low altitude populations. Interestingly, our results diverge from those of Chun et al. [47] who identified lower phenotypic structure for height and biomass across study populations. In our work, we however sampled populations across steeper climatic gradients, and hence capture more phenotypic variation related to the adaptation to abiotic conditions which can explain why we also detect higher P_ST_ values. Moreover, Hodgins et al. [48] also showed high genetic differentiation in life-history traits between native and introduced populations of common ragweed along quite large climatic gradients in Europe, notably for growth, biomass and plant width, thus supporting our conclusions.

The combined analysis of genetic, functional and niche data tend to support a scenario of rapid climatic adaptation of *A*. *artemisiifolia* during its colonization of the French Alps. Pre-introduction adaptation is unlikely to explain our results as, based on the estimation of its global climatic niche, the most adverse climatic conditions encountered in the study region have apparently never been experienced anywhere else by the species, including in other adventive regions. Our results also show that isolation by distance, and allele surfing cannot explain the genetic clines observed along the species niche gradients. Such rapid adaptation to alpine conditions has probably happened within the last decades where an increasing number of populations have been recorded at higher elevations. This process may have been fostered by its history of multiple introductions and intense human mediated dispersal, which likely increased intra-population genetic diversity in the region (as shown by Chun et al. [49]). This may have allowed novel genetic recombination and permitted the colonization of colder climates in higher locations.

### Genetically based climatic niche expansion

To evaluate whether local adaptation has permitted niche expansion, we used an innovative stratified sampling-design based on the combination of the climatic niches of the species estimated at both global and regional scales. This allowed us locating populations currently experiencing novel climatic space compared to the known species' worldwide climatic niche. This feature is obviously dependent on the ability of the climatic niche estimations at two scales to correctly detect populations occurring out of the species' global niche (S1 Appendix). In highly heterogeneous environments, a model calibrated at the global scale could indeed fail to fully capture the environmental variability that is observable at the regional scale, thus confounding niche estimates. Nevertheless, the fact that the populations suspected of adaptation show a clear signal of genetic adaptation (both from outlier markers and phenotypic traits) comforts us with the robustness of our sampling design. Altogether, our results converge in suggesting that (1) forefront populations experiencing more stressful alpine conditions show clear molecular signatures of selection, (2) that these selected markers correlate to phenotypic traits related to climatic niche adaptation, and that (3) all these patterns cannot be expected from pure drift and migration alone. This tends to demonstrate that *A*. *artemisiifolia* is currently adapting to adverse climatic conditions of alpine environments. Hence, rapid local adaptation and climatic niche expansion of such invasive species is likely to enhance its invasion rate [50].

Perhaps an important finding for predicting future spread of this species in alpine environments is that forefront (alpine) populations harbor lower genetic diversity and reduced phenotypic variation than core (low elevation) populations, as expected following sequential founder effects during colonization [14]. Indeed, this apparent depletion of evolutionary potential of forefront populations, as measured by selection skewers, seems to be due to a reduction in the phenotypic variance for individual biomass, which is main driver of phenotypic integration across study populations (i.e. highly correlated with *Pmax* of ***G*** matrices). Thus, the species may have a reduced capacity to adapt toward colder temperatures and lower levels of solar radiation, which may slow down its future niche and range expansion. Further empirical and theoretical simulations studies are, however, needed to prove this true, and estimate the ultimate limits to niche expansion in this invasive species.

It might appear surprising that populations at niche edges show continuous adaptation towards more alpine climatic conditions, especially despite evidence for genetic bottlenecks; however such unexpected situation has already been reported for other invasive plants [6]. Two mechanisms could explain this result. First, the relative genetic isolation of edge populations from central populations (as exampled by their higher F_ST_ values) could favor local adaptation by limiting gene swamping from central populations. Second, in some conditions genetic bottlenecks have the potential to maintain certain levels of genetic variance due to the change in epistatic effects or decreased dominance effects [51]. Testing this hypothesis will require the use of co-dominant markers to analyze progenies produced in natural conditions (e.g., measuring progenies kinship), in order to better understand the origin of genetic variation in phenotypic traits.

### Potential limitations and perspectives

Although our results indicate that *A*. *artemisiifolia* is likely adapting to novel climatic conditions, two main limitations remain in our approach: the measure of genetic variance in phenotypic traits and possibly confounding maternal or epigenetic effects.

The first limitation lies in the degree of relatedness of individuals that we did not measure. We assumed that most of the phenotypic variance was due to genetic additive effects and that epistatic variance was negligible. We did so because *A*. *artemisiifolia* is strictly self-incompatible and shows consistently high outcrossing rates throughout its native and invasive ranges, irrespectively of population density [21,22]. Since it produces massive amounts of wind-dispersed pollen, we reasonably assumed that a similar pollen pool -uniform at the population scale- has pollinated all mother plants leading to a constant genetic co-ancestry within all progenies. Since the environment was constant across our common garden experiment, we then considered that the genetic variance retained by different mother plants scales with population-level genetic variance in phenotypic traits. Additionally, because in general dominance and epistatic gene interaction are likely to reduce Q_ST_ [52,53], and because fitness-related traits (such as height and biomass) tend to contain greater non-additive genetic variance than do non-fitness related traits [54], we can expect our Q_ST_-F_ST_ comparison to be rather conservative. Our jackknife procedure confirmed that our relatively low number of mother plants still allowed us to get reasonable estimate of genetic variance (Table C and Fig G in S2 Appendix). Nonetheless, further pedigree estimations or crossing experiments would bring more insights into spatial patterns of genetic variance in phenotypic traits. Additionally, replicating common gardens in different environments would also be an interesting experimental complement to our study to test for genotype-environment interactions. However, the present study aimed at estimating quite large scale genetic variance across populations, based on the sampling of 115 mother plants and the growing of 1921 plants in common garden conditions. Thus, any experimental extension would have to be based on a specific subset of well-chosen populations.

The second limitation concerns the use of first generation seeds potentially leading to maternal or epigenetic effects that may have inflated our estimates of population differentiation and heritability for the different traits. Using proxies to test for maternal effects (see Fig E in S2 Appendix), we found no significant effects, suggesting that maternal effects may have been low or negligible in our experiment. Regarding epigenetic effects, their impacts on the results depend on their durability. If they are relatively stable over time then it is difficult to distinguish them from “pure” genetic variability. If they are relatively quickly reversible we could detect that the pattern changes over time (as for classical maternal effects). However, experimental limitations due to the highly allergenic pollen did not allow us to test for temporal changes in our estimates of population differentiation or trait heritability over multiple generations. Nonetheless, the fact that we found trait-marker associations suggests that epigenetic effects may not alone explain all spatial patterns of phenotypic variation, and that a significant part of phenotypic differences observed in natural populations can still be attributed to genetically-based adaptation. One promising research direction will be the use of transcriptomes to look for candidate genes and explore the functional aspect of local adaptation (as suggested by Hodgins and colleagues [55] on a promising example on *A*. *artemisiifolia*). Alternatively, QTL or GWAS procedures would also further reveal the genetic basis of niche expansion in this species.

## Conclusions

Here we provided a case study on how the combination of distribution modeling, field study and molecular and experimental work can offer essential glimpses into the nature of adaptive constraints during species invasions. The inference of species climatic niche at both global and regional scales proved to be a crucial approach to identify potential niche expansion and to pinpoint populations suspected of on-going adaptation. The combined study of allele association with environment and phenotypic traits also provided valuable insights into the nature of natural selection imposed by niche expansion during colonization of a new climatic space. Thus, our results suggest that over the course of last decades, *A*. *artemisiifolia* may have been expanding its niche toward more stressful alpine conditions, but that this expansion will probably be slowed down in the future due to functional trait correlations and depleted adaptive genetic variation. Such results suggest that some species' climatic niches could evolve on very short timescales, and can thus be very labile ecological characteristics in some short-lived invasive organisms. Repeating this type of study would be now interesting to better understand under which conditions niche evolution can significantly impact species' range expansion, and better explore the population mechanisms of niche evolution.

## Supporting Information

S1 Appendix Details on the methods. These details include niche estimation and niche comparison methods, AFLP protocols, population genetic indices and spatial structure methodology, as well as common garden protocol.
(DOC)

S2 Appendix Details on the results. These details include allele-environment tests, population genetic structure, experimental design and maternal effects, trait-niche gradient regression models, and ***G*** matrix analysis.
(DOCX)
