# Supplementary material for: Is There Any Evidence for Rapid, Genetically-Based, Climatic Niche Expansion in the Invasive Common Ragweed?
Source: PLoS One. 2016 Apr 26;11(4):e0152867. doi: 10.1371/journal.pone.0152867 (PMC4846088; doi:10.1371/journal.pone.0152867)
Supplement: S1 Appendix — These details include niche estimation and niche comparison methods, AFLP protocols, population genetic indices and spatial structure methodology, as well as common garden protocol. (DOC) [file pone.0152867.s001.doc]

**Appendix S1: Details on the methods**

The methodological framework includes three main steps: (1) multiple scale climatic niche estimations and comparison, (2) population genetic analyses, and (3) quantitative traits analyses (Fig A).

**Regional Niche Estimation.** The regional niche for the French Alps was built using 3,888 observations of *A. artemisiifolia* (source CBNA) and 10,000 background data randomly selected in the study region. Because we wanted to both: stratify our sampling with the regional niche model, and provide an ecological understanding of the evolutionary processes occurring along the regional niche gradients, we needed to use a restricted number of environmental variables to estimate this niche. To do so we selected two variables from a set of 21 topo-climatic variables obtained from the meteorological model Aurelhy (see Table A), based on interpolated measurements at a resolution of 100 m summarizing climatic information over 1971 and 2000 [1]. The selection was based on a principal component analysis (PCA), selecting the two variables that best co-varied with the two first axes of the PCA, and which had a coefficient of correlation lower than 0.5 (Spearman non-parametric coefficient of correlation). The two variables that most segregated the sites occupied by the species in the French Alps: mean annual solar radiation and mean summer temperature (highly correlated with first two axes of the PCA and explaining 70% of the inter-sites differences). Based on the same ensemble modelling as for the global niche estimation we confirmed that the regional model was well performing (AUC=0.94) [2].

**Global and Regional Niche Comparison.**

In this framework comparing global and regional niches, it is important to note two key aspects: (1) the detection of locally adapting population is made via a conservative test, and (2) the use of different niches estimated with different variables at different scales is an advantage.

First, the use of all occurrences of the species across the world allows the estimation of the full range of environmental conditions under which the species can survive, and thus approximates the “true” physiological limitations of the species, that is its fundamental niche [3]. It can be noted that, by combining populations from the native range as well as from other invaded regions there are chances that the dataset contains some: (i) populations that are adapting to local climatic conditions (though unlikely as shown by [4]), as well as (ii) sink populations. However, the addition of such populations into the global niche estimate should just enlarge the global niche edges, and make the detection of locally adapting populations in the region of interest less likely. Hence we believe that our global niche measure is more over-estimated than under-estimated, and thus our test and experimental design is conservative.

Second, because we worked at two different geographical extents (global *vs.* regional) we also had occurrence data at two spatial resolutions (2.5km *vs.* 100m). Even though we could have upscaled the regional data to a resolution of 2.5km to use the same climatic variables at both resolutions, we decided to keep with the finest resolution at the regional scale (i.e. 100m) to be able to use more ecologically meaningful variables. We however tested that the populations suspected of rapid adaptation remained outside of the global niche even when considering the coarse global climatic resolution variables (Fig B). It should also be noted that even though it is not possible to compare the two niches (global *vs.* regional) in a niche space (because they do not rely on the same environmental variables), it remains possible to project these niches in geographical space and then identify populations that are predicted “outside of the global niche” and at the same time “within the regional niche”: the populations that we suspect of adaptation to novel climate.

It can be noted that the method published by Petitpierre et al [4] is somehow similar to ours, but has not been developed to really identify populations rapidly adapting to new environmental conditions. Based on a former work [5], we in fact go in greater detail than Petitierre et al [4] by comparing global vs. regional niches vs. regional occurrences (and not only native and invaded niches). Indeed, by not only using occurrences of the species in its native range, but rather all occurrences available in the globe (i.e. including all other invaded regions) we are more likely to detect niche shifts that are actually due to new adaptations to new environmental conditions, instead of just changes in the local biotic conditions for example (e.g. presence of different competitors or mutualistic partners).

**Amplified Fragment Length Polymorphism (AFLP) Protocol.** Genomic DNA was extracted using DNeasy 96 Plant kit (Qiagen©) according to the manufacturer’s instructions for 10 individuals in each of the 27 populations (Table B). Three blanks and 12 repetitions (~5%) were included to further assess the repeatability and reliability of the markers. We used an Amplified Fragment Length Polymorphism (AFLP) protocol based on standard methods [6] with minor modifications. Digestion of total DNA was performed together with ligation of double stranded adaptors for 4h at 37 °C in a 11µl mix using 1U of MseI, 5U of EcoRI (New England Biolabs), 1U of T4 DNA ligase (Roche and its associated buffer), 50mM NaCl and 50ng/µl BSA. Products were diluted 1:10 and the preselective amplification (120s at 72°C; 25 cycles 30s at 94°C, 30s at 56°C, 120s at 72°C; with a final elongation 10min at 72°C) was carried out in a 25µl volume containing 5pmol of the *Eco*RI+A primer, 5pmol of the *Mse*I+C primer, 200μM of each dNTP, 1.5 mM MgCl_2_, 1X PCR Buffer II pH 8.3, 0.5U Ampli*Tag* Gold DNA polymerase (Applied Biosystems) and 3μL of diluted digestion-ligation reaction. After a 1:20 dilution of preselective PCR products, selective amplifications were conducted with three primer combinations: *Eco*RI+ACG / *Mse*I+CAA, *Eco*RI+ACT / *Mse*I+CTG and *Eco*RI+AGC / *Mse*I+CTG. *Eco*RI+3 primers were labelled with 6-carboxy-fluorescein (6-FAM). Each selective amplification reaction contained 2.5pmol of each primer, 200μM of each dNTP, 2.5mM MgCl_2_, 1× Taq polymerase buffer, 8ng/μL BSA, 0.5U Ampli*Tag* Gold DNA polymerase (Applied Biosystems) and 2.5μL of diluted preselective amplification reaction, in a final volume of 12.5μL. We used the following PCR conditions: 95°C for 10min; 13 cycles of 30s at 94°C, 60s at 65-55°C, and 60s at 72°C; 23 cycles of 30s at 94°C, 60s at 56°C, 60s at 72°C; and a final elongation of 10min at 72°C. PCR products were purified using columns of half to half 5% Sephadex G50 and Sephacryl S200. Finally, 1.5µl of the diluted FAM labelled products were mixed with 10µl of HiDi formamide and 0.1µl Genescan ROX 500 size standard (Applied Biosystems), and electrophoresed on an ABI PRISM XL 3130 capillary sequencer.

In order to locate genotyping errors or contamination issues, we routinely included replicated samples (DNA extractions) within and between plates during molecular biology analyses. We adopted a strict sample arrangement on 96-well plates [7], and included two replicates among plates, two replicates within plates, and additional 5–10% blind replicates (labeled as X). When inconsistency was found between replicates, plates were automatically discarded from all analyses.

Allele scoring was performed with PeakScanner© v2.0 (Applied Biosystems) and RawGeno [8] with a visual checking of the bins. We used R v.2.15.0 software (R Development Core Team 2012) to remove peaks occurring in the blank samples and the ones not consistently present between replicates. The initial observed error rate was 5% (i.e. the reproducibility was 95%) and during the cleaning the mismatch error rate acceptance was of 0%. Peak size outliers were also removed, as well as singleton peaks and ubiquitous ones. Following this stringent cleaning procedure, a set of 240 AFLP markers was kept for analyses.

**Fis, Fst and He Calculations.** To calculate the inbreeding coefficient F_IS_ and the degree of population differentiation F_ST_, which are not directly available for dominant markers such as AFLPs, we used BAYESCAN v.2.1 [9]. The method infers the proportion of heterozygote individuals based AFLP band intensities. BAYESCAN assumes that all sampled populations stem from an ancestral population according to the F-model [10]. For instance, population F_ST_ are the estimated difference between the focal population and the *ancestral* population. F_IS_ and F_ST_ statistical significance were tested using the randomization procedure with 20 pilot runs, 5000 iterations, 50,000 burn in and 50,000 of thinning interval [9,11]. To compare with recent studies, a global F_ST_ for all populations was also generated with AFLP-surv v.1.0 (with 9,999 randomization, choosing the non-uniform prior distribution and an average F_IS_ estimated from BAYESCAN analysis). For each population the genetic diversity H_e_ [12] was also calculated with AFLP-surv v.1.0 (with 9,999 randomization, choosing the non-uniform prior distribution and an average F_IS_ estimated from BAYESCAN analysis) [13].

**Spatial Genetic Structure.** To investigate the genetic structure of the populations we used the Bayesian clustering program STRUCTURE 2.3 [14] from which we extracted the optimal number clusters. This method determines the more likely number of genetic clusters from the dataset and assigns to each individual a probability of belonging to these clusters. Given the potential high gene flows in anemophilous annuals such as common ragweed, we used the admixture model, using the LocPrior option [15] and assumed correlated allele frequencies across populations [10,14]. We run STRUCTURE for 1,000,000 iterations after a burn-in of 500,000 iterations, and repeated the procedure 5 times for each K value from *K*=1 to *K*=20 (*K* being the number of clusters). The most parsimonious number of cluster was identified using the log-likelihood curve, and we chose the one at the beginning of the log-likelihood plateau. For the most likely number of clusters (K=4) we performed five more runs, averaged the ten runs using CLUMPP v.1.1.2 [16], and displayed it using the software DISTRUCT v.1.1 [17].

**Common Garden Protocol.** The seeds collected during fall 2010 in 18 different populations (Table B) were weighted and dried at room temperature and then stratified at 4°C during one month. The dormancy of all seeds was broken at the same time (12^st^ April 2011) and germination condition were of 20/10°C 12h/12h light/dark conditions per day (daily permutation of seed family position in the germination room to avoid position effect; following the recommendations of [18]). The germinated seeds were then sown in 10x10x20 cm^3^ pot size containing a mixture of 1/3 sand, 1/3 compost soil and 1/3 row soil. These pots were placed for the first 15 days under a canvas for solar radiation protection in order to limit transplantation shock, and then randomly placed into 3 rows of 10 blocks containing each about 100 individuals. Throughout the experiment the plants were watered until soil saturation every two days.

Right before flowering onset (ca. 2.5 months of growth), plants were collected to measure four quantitative traits: the total dry biomass (above + below ground biomass), the plant height, the shoot-root dry biomass ratio, and the leaf dry matter content (LDMC, leaf dry mass/fresh mass). To do so, five mature leaves were harvested on each individual plant and weighted to measure their fresh biomass, and roots were washed into water pots. All plant compartments were dried separately at 60°C for 48h and then weighted. Note that given the large size of the experiment, only half of the plants were randomly chosen to be measured (ca. 1134 measured plants).

It can be noted that traits associated with the phenology of *A*. *artemisiifolia* would have been highly interesting to estimate the level of local adaptation to the length of the growing season that is shorter at high elevations. However, we decided to not measure them for two reasons: we wanted to collect the traits for all individuals at the same moment (including destructive traits such as total plant biomass), and we were not allowed to pursue the common garden experiment as soon as the first flower was blooming (to avoid pollen spread).

There is unfortunately no information available about the age of the populations, as well as potential sources of introductions.

**REFERENCES**

1. Benichou P, Le Breton O. Prise en compte de la topographie pour la cartographie des champs pluviométriques statistiques. La Météorologie. 1987;7: 23–34.

2. Pearce J, Ferrier S. Evaluating the predictive performance of habitat models developed using logistic regression. Ecol Modell. 2000;133: 225–245.

3. Gallien L, Münkemüller T, Albert CH, Boulangeat I, Thuiller W. Predicting potential distributions of invasive species: Where to go from here? Divers Distrib. 2010;16: 331–342. doi:10.1111/j.1472-4642.2010.00652.x

4. Petitpierre B, Kueffer C, Broennimann O, Randin C, Daehler C, Guisan A. Climatic niche shifts are rare among terrestrial plant invaders. Science. 2012;335: 1344–8. doi:10.1126/science.1215933

5. Gallien L, Douzet R, Pratte S, Zimmermann NE, Thuiller W. Invasive species distribution models - how violating the equilibrium assumption can create new insights. Glob Ecol Biogeogr. 2012;21: 1126–1136. doi:10.1111/j.1466-8238.2012.00768.x

6. Vos P, Hogers R, Bleeker M, Reijans M, van de Lee T, Hornes M, et al. AFLP: a new technique for DNA fingerprinting. Nucleic Acids Res. 1995;23: 4407–14.

7. Bonin A, Bellemain E, Broken Eidesen P, Pompanon F, Brochmann C, Taberlet P. How to track and assess genotyping errors in population genetics studies. Mol Ecol. 2004;13: 3261–3273. doi:10.1111/j.1365-294X.2004.02346.x

8. Arrigo N, Tuszynski JW, Ehrich D, Gerdes T, Alvarez N. Evaluating the impact of scoring parameters on the structure of intra-specific genetic variation using RawGeno, an R package for automating AFLP scoring. BMC Bioinformatics. 2009;10: 33. doi:10.1186/1471-2105-10-33

9. Foll M, Fischer MC, Heckel G, Excoffier L. Estimating population structure from AFLP amplification intensity. Mol Ecol. 2010;19: 4638–47. doi:10.1111/j.1365-294X.2010.04820.x

10. Falush D, Stephens M, Pritchard JK. Inference of Population Structure Using Multilocus Genotype Data: Linked Loci and Correlated Allele Frequencies. Genetics. 2003;164: 1567–1587. Available: http://www.genetics.org/content/164/4/1567.full

11. Fischer MC, Foll M, Excoffier L, Heckel G. Enhanced AFLP genome scans detect local adaptation in high-altitude populations of a small rodent (Microtus arvalis). Mol Ecol. 2011;20: 1450–62. doi:10.1111/j.1365-294X.2011.05015.x

12. Nei M. Molecular evolutionary genetics. New York: Columbia University Press; 1987.

13. Vekemans X, Beauwens T, Lemaire M, Roldan-Ruiz I. Data from amplified fragment length polymorphism (AFLP) markers show indication of size homoplasy and of a relationship between degree of homoplasy and fragment size. Mol Ecol. 2002;11: 139–151. doi:10.1046/j.0962-1083.2001.01415.x

14. Pritchard JK, Stephens M, Donnelly P. Inference of population structure using multilocus genotype data. Genetics. 2000;155: 945–959. doi:10.1111/j.1471-8286.2007.01758.x

15. Hubisz MJ, Falush D, Stephens M, Pritchard JK. Inferring weak population structure with the assistance of sample group information. Mol Ecol Resour. 2009;9: 1322–1332. doi:10.1111/j.1755-0998.2009.02591.x

16. Jakobsson M, Rosenberg NA. CLUMPP: a cluster matching and permutation program for dealing with label switching and multimodality in analysis of population structure. Bioinformatics. 2007;23: 1801–6. doi:10.1093/bioinformatics/btm233

17. Rosenberg N a. DISTRUCT: A program for the graphical display of population structure. Mol Ecol Notes. 2004;4: 137–138. doi:10.1046/j.1471-8286.2003.00566.x

18. Willemsen RW. Effect of stratification temperature and germination temperature on germination and induction of secondary dormancy in common ragweed seeds. Am J Bot. 1975;62: 1–5.

**Figure A.** **Schematic representation of methods used in this study as well as their links.** The analyses were performed following three steps: (1) multiple scale niche estimations and comparison, (2) population genetic analyses, and (3) quantitative traits analyses. Note that some analysis involved the use of the outcomes of former steps, here represented by doted arrows.

**Figure B**. **Representation of the *A. artemisiifolia*’s presences on the environmental space.** The figures represent the 1^st^ and 2^nd^ axis of Principal Component Analysis (PCA) made on the species presence and the 5 climatic variables used to estimate the global niche (2.5km resolution). In the panels (a) and (b) the worldwide species occurrences are represented in black (i.e. the GBIF database), and in panel (b) the regional occurrences are in red (i.e. French Alps database). In the panel (c) and (d), we can see the density of the worldwide species occurrences in a colour gradient, and in panel (d) the regional occurrences in red. In the last panel (e), we show on a map all occurrences that have been used to build the global niche of the species (4803 occurrences). The populations of the native range are represented by green dots (1293 occurrences), and the ones of the invaded ranges are represented by red dots.

| Topography | Elev_range | Elevation range within the pixel |
| --- | --- | --- |
|  | Slope | Slop within the pixel |
|  | Aspect | Orientation of the pixel (in degrees from the North directions) |
|  | twi | Topographic wetness index (based on the concavity or convexity of the area) |
| Growing period | GS_length | Length of the growing season (in days) |
|  | Ddeg_556 | Number of degree days (above 5.56°C) |
| Temperature | T_sd | Temperature standard deviation over the year |
|  | Tave_678 | Mean temperature of the warmest season (June-July-August) |
|  | Tave_1202 | Mean temperature of the coldest season (December-January-February) |
|  | T_max_0112 | Maximal temperature of the year |
|  | T_min_0112 | Minimal temperature of the year |
|  | T_max_1202 | Maximal temperature of the coldest season (December-January-February) |
|  | T_min_678 | Minimal temperature of the warmest season (June-July-August) |
| Solar radiation | Srad_0112 | Annual solar radiation |
|  | Srad_678 | Mean solar radiation of the warmest season (June-July-August) |
| Moisture | M_ind_sd | Moisture index standard deviation over the year |
|  | M_ind_678 | Moisture index average during the warmest season (June-July-August) |
| Precipitation | Prcp_sd | Precipitation standard deviation over the year |
|  | Prcp_1202 | Annual level of precipitation |
|  | Prcp_0112 | Precipitation during the coldest season (December-January-February) |
|  | Prcp_678 | Precipitation during the warmest season (June-July-August) |

**Table A.** **List of the 21 variables tested to identify the best drivers of *A. artemisiifolia’s* presences and absences at the regional scale**. The two variables highlighted in brown are the ones selected via a PCA.

| **PopID** | **X** | **Y** | **Tave_678** | **Srad_0112** | **In/Out global niche** | **In Common Garden?** |
| --- | --- | --- | --- | --- | --- | --- |
| 7 | 924225 | 1946682 | 17.081 | 4.797 | *Out* | No |
| 8 | 924947 | 1945925 | 17.302 | 6.996 | *Out* | Yes |
| 23 | 862392 | 2020645 | 18.954 | 9.369 | In | Yes |
| 25 | 862195 | 2023648 | 18.588 | 8.922 | In | Yes |
| 27 | 862644 | 2025055 | 19.266 | 9.768 | In | Yes |
| 33 | 881899 | 2017800 | 17.937 | 8.653 | *Out* | Yes |
| 34 | 879936 | 2015275 | 18.246 | 7.066 | *Out* | Yes |
| 36 | 874004 | 2012055 | 18.992 | 7.761 | In | Yes |
| 37 | 869084 | 2013186 | 18.515 | 9.014 | In | Yes |
| 42 | 871507 | 2022895 | 17.977 | 10.451 | In | Yes |
| 50 | 833330 | 2014249 | 19.986 | 9.242 | In | Yes |
| 56 | 838583 | 2011483 | 19.438 | 6.985 | In | Yes |
| 58 | 838551 | 2012857 | 18.881 | 9.557 | In | Yes |
| 60 | 841593 | 2017784 | 15.845 | 10.446 | *Out* | No |
| 63 | 854644 | 2018996 | 15.572 | 9.489 | *Out* | No |
| 89 | 828547 | 2078177 | 19.438 | 10.303 | In | Yes |
| 97 | 799364 | 1958666 | 21.133 | 9.902 | In | No |
| 102 | 806392 | 1905795 | 22.299 | 9.287 | In | Yes |
| 105 | 858229 | 2038535 | 19.917 | 8.928 | In | Yes |
| 110 | 847109 | 2058452 | 17.979 | 9.201 | In | Yes |
| 112 | 837759 | 2067973 | 18.755 | 10.842 | In | No |
| 120 | 855976 | 2038733 | 19.888 | 6.742 | In | Yes |
| 138 | 842957 | 2103447 | 17.630 | 7.652 | In | No |
| 139 | 843473 | 2103871 | 17.879 | 7.625 | In | Yes |
| 151 | 875015 | 2048070 | 16.750 | 6.834 | *Out* | No |
| 191 | 774826 | 2069258 | 17.333 | 11.106 | In | No |
| 198 | 811822 | 2072937 | 19.352 | 9.293 | In | No |
| *Common Garden* | 895600 | 1959400 | 17.593 | 9.041 | In | -- |

**Table B. Description of the populations sampled for genetic analyses and the common garden experiment.** Coordinates (in NTF France zone II coordinate system) and environmental condition of the 27 populations used for AFLP analysis, as well as the coordinates and environmental conditions of the common garden. The 6^th^ column indicates whether the populations are located outside or within the global niche estimate for the species (i.e. suspected of adaptation to new environmental conditions or not). The 7^th^ column indicates which populations were used in the common garden experiment.
