# Supplementary material for: Is There Any Evidence for Rapid, Genetically-Based, Climatic Niche Expansion in the Invasive Common Ragweed?
Source: PLoS One. 2016 Apr 26;11(4):e0152867. doi: 10.1371/journal.pone.0152867 (PMC4846088; doi:10.1371/journal.pone.0152867)
Supplement: S2 Appendix — These details include allele-environment tests, population genetic structure, experimental design and maternal effects, trait-niche gradient regression models, and G matrix analysis. (DOCX) [file pone.0152867.s002.docx]

**Appendix S2: More details on the results**

**Controlling for Multiple Tests of the Allele-Environment Relationships.** In order to test whether some markers exhibited putative selection signatures related to the two main regional niche gradients (temperature and radiation) we used logistic regressions. Doing so we tested for a significant association (linear and/or quadratic) of each marker with temperature, radiation and their interaction term (e.g., [1]) following a stepwise procedure based on AIC. Then, for the markers associated to the environmental factors, we tested whether the residuals were spatially-autocorrelated using Moran’s I (R package *spdep* [2]) but none was significant.

To estimate the background rate of false discovery due to our data, we also controlled for multiple testing. Indeed, because the probability of finding significant relationships between the allelic frequencies and environmental variables can increase when the number of test is high, it is necessary to test whether the observed relationships are not obtained by chance only. To do so, we first applied a 999-randomisation procedure on the presence of each marker independently. That is to say, for each marker, we permuted its allele presences across the samples and tested whether we still found significant relationships with the environmental gradients. Doing so we found that detecting 36 significant relationships is significantly higher than what one could expect by chance (Fig C). Second, for drawing interpretations about potential molecular signatures of climatic adaptations and allele-traits association, we only kept the 29 markers that overall showed a level of false discovery rate under the 5% threshold, using the procedure described by Verhoeven et al*.* [3].

**Population Neutral Genetic Structure.** When looking at the Fig D it can be noted that in the populations 33, 63 and 138, individuals were assigned to cluster 3 with high assignment probabilities (on average 43 %). Populations 7 and 8, located apart in South East of the sampling region, were assigned distinctly to respectively cluster 1 (59% on average) and cluster 4 (56% on average) with only 38% of assignment probability for cluster 2. These populations however did not present particularly extreme values of H_e_, F_IS_ nor F_ST_ (Table E). Such result suggests that the assignment to the cluster 1 and 4 is not due to genetic drift in the populations 7 and 8, but more likely due to admixture with other genetic pools that are not present in the region, or at least not detected (or represented) in the sampled populations.

**Testing the Experimental Design Effects.** The block design effect was tested using a generalized mixed model likelihood ratio test (with Restricted Maximum Likelihood and a bootstrap test) between the full (Eq. S2.1) and all possible reduced models (R package *lme4* [4])*.* The random effects of population and family provenance were always included, along with block-design effect when significant.

Trait ~ 1 + 1|Population + 1|Population/Family + 1|ExperimentalDesign (Eq. S2.1)

The common garden was composed of three “main blocks” (hereafter called *M-blocks*), sub-divided into ten “smaller blocks” (hereafter called *S-block*). For each trait measured at the end of the experiment, these two block effects were tested to detect whether they have a significant influence on the measurements. The results are presented in Table B.

**Testing for Maternal Effects.** It is important to keep in mind that due to its highly allergenic pollen, obtaining a second generation of *A. artemisiifolia* in common garden or greenhouse conditions is not doable: strong health issues for experimental workers and surroundings have to be anticipated. Hence our common garden experiment potentially includes maternal effects. To test whether maternal effects could plague our analyses, we first estimated the coefficient of genetic variation (CV_G_, measured as V_G_/trait mean [5]) for the plant height measured at different times of development as suggested by Petit *et al.* [6]. If maternal effects occur, we expect them to be more important at juvenile stages and to decrease over time. On the contrary, we found that traits CV_G_ increased over time (Fig E), indicating that the maternal effect was probably negligible. Secondly, we also tested whether the inclusion of seed mass as a covariable of the trait-environment regressions significantly affected our results, as seed mass is thought to capture part of maternal effects. We found no significant differences. Overall, we found little potential influence of maternal effects on traits measured in common garden.

**Q_ST_ Estimations.** For the four studied traits Q_ST_ was calculated as *V_pop_*/(*V_pop_* + 2**V_G_*), where *V_pop_* is the trait variance inter-population and *V_G_* the additive genetic variance component. *V_G_* was calculated as *N***V_fam_*, where *V_fam_* is the inter-family trait variance within populations and *N* is the degree of relatedness between individuals within families. We considered individuals of the same seed family to be either half- or full-sibs, that is *N*=4 or N=2, due to the outcrossing mating system of the species but high inbreeding within populations (Table E). Confidence intervals, showing Q_ST_ estimates robustness to the low family sample size, were calculated via a jack-knife resampling procedure over the seed families (Table C). Then, when comparing species Q_ST_ and F_ST_, Q_ST_ < F_ST_ is usually considered as an evidence for stabilizing selection, while Q_ST_ > F_ST_ may indicate divergent selection ([7] but see [8] for limits to this approach).

**Trait - Niche Gradients Regressions.** We tested whether the traits changed over the regional niche of the species using generalized linear mixed models (R package *nlme* [4]). Both temperature and radiation were included in simple and quadratic forms as fixed effects as well as their interactions, along with population, family and block-design random effects (as in Eq. S2.1). Each combination of fixed effect parameters was tested and for each trait we retained the model with the lower AIC score (∆AIC representing the AIC difference with the intercept models, Table D). Additionally, we tested whether these models were significantly better than the intercept models using F-tests (Table D).

***G* matrices and *Pmax* Direction along the Niche Gradients.** To build the ***G*** matrices for each population we used MANOVAs as they can incorporate a family effect and thus take into account the fact that all individuals belonging to one family are not independent (code modified from [9]). The ***G*** matrices were calculated separately for each population, using 6 families per population. Note that all traits were centred and scaled prior analysing. Then for each population we estimated *Pmax*’s direction (i.e. the direction of the first eigenvector of ***G***), which is particularly interesting for comparing different populations, where the difference between populations is estimated as the cosine between their *Pmax*. For the direction of *Pmax*, we tested whether populations from similar climate presented similar *Pmax* directions, using a correlogram (including 9999 matrix randomizations and Bonferroni correction for multiple testing with the R package *vegan* [10]; Fig F). We also assessed the influence of the low number of sampled seed families on ***G*** matrix estimates via a jack-knife resampling procedure over seed families (Fig G).

**Selection Skewer Method (SSM).** SSM is an extension of the Random Skewers Method [11], which compares the response of different ***G*** matrices to a selection vector (with a given intensity of selection) to test whether they will result in alternative evolutionary responses. We choose to apply one selective scenario on the populations: a decrease of temperature and of solar radiations, to test whether populations at the niche edge can pursue adaptation toward more stressful conditions. Note that we applied this method only to the populations occurring in the colder half of the regional niche, as these populations are the ones potentially contributing to niche expansion

**REFERENCES**

1. Manel S, Albert CH, Yoccoz NG. Sampling in landscape genomics. 2012;888: 3–12.

2. Cliff AD, Ord JK. Spatial autocorrelation. London: Pion; 1973.

3. Verhoeven KJF, Simonsen KL, McIntyre LM. Implementing false discovery rate control: increasing your power. Oikos. 2005;108: 643–647. doi:10.1111/j.0030-1299.2005.13727.x

4. Pinheiro JC, Bates DM. Mixed-effects models in S and S-Plus. Chambers JM, Eddy  W. WH, Sheather S, Tierney L, editors. Statistics and Computing. New York: Springer-Verlag; 2000.

5. Houle D. Comparing evolvability and variability of quantitative traits. Genetics. 1992;130: 195–204.

6. Petit C, Freville H, Mignot A, Colas B, Riba M, Imbert E, et al. Gene flow and local adaptation in two endemic plant species. Biol Conserv. 2001;100: 21–34.

7. Merilä J, Crnokrak P. Comparison of genetic differentiation at marker loci and quantitative traits. J Evol Biol. 2001;14: 892–903. doi:10.1046/j.1420-9101.2001.00348.x

8. Whitlock MC. Evolutionary inference from QST. Mol Ecol. 2008;17: 1885–1896. doi:10.1111/j.1365-294X.2008.03712.x

9. Martin G, Chapuis E, Goudet J. Multivariate Qst-Fst comparisons: A neutrality test for the evolution of the G matrix in structured populations. Genetics. 2008;180: 2135–2149. doi:10.1534/genetics.107.080820

10. Dixon P. VEGAN, a package of R functions for community ecology. J Veg Sci. 2003;14: 927–930. doi:10.1111/j.1654-1103.2003.tb02228.x

11. Revell LJ. The G matrix under fluctuating correlational mutation and selection. Evolution. 2007;61: 1857–72. doi:10.1111/j.1558-5646.2007.00161.x

**Figure C.** **Observed number of alleles showing a significant relationship with environment, and expected rate of detection errors**. The white histogram represents the number of significant relationships obtained by random assignments of the allele presences across the individuals, and the red line indicates the observed number of allele-environment relationships.

**Figure D**. ***Ambrosia artemisiifolia* population genetic structure.** The histogram represents the complete assignment probabilities of each individual to the K = 4 clusters defined by STRUCTURE.


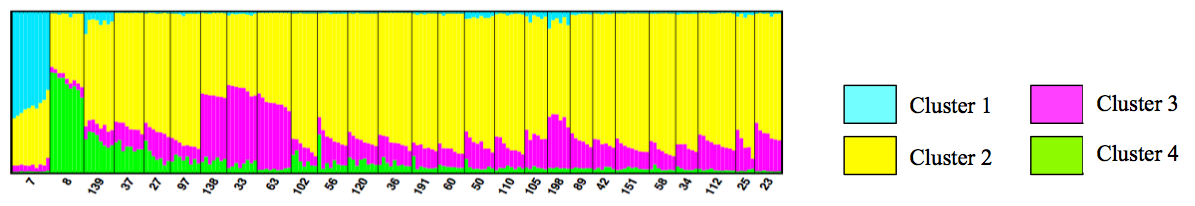


**Figure E.** **Indirect test for maternal effects in the common garden experiment**. Representation of the evolution of the genetic coefficient of variation (CV_G_) for the plant height over time during the common garden experiment.


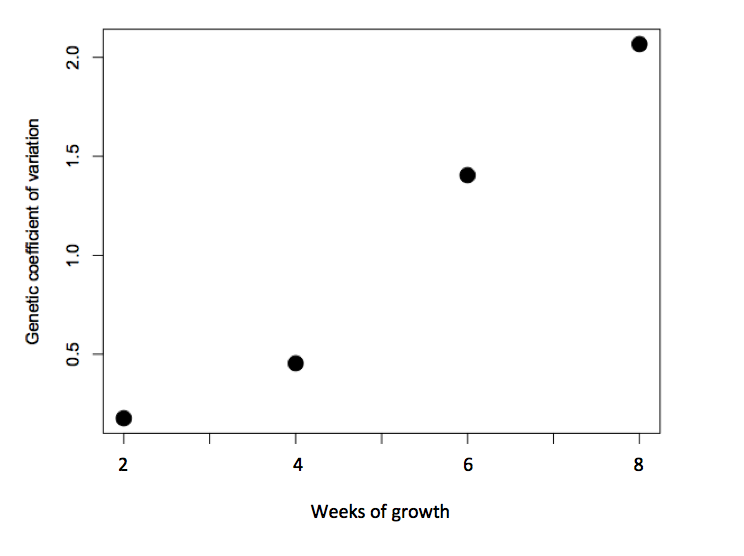


**Figure F.** **The direction of the phenotypic integration between populations is more similar in resembling environmental conditions**. Mantel correlogram (using the R package *vegan*) representing the differences in *Pmax* directions across populations and how they vary according to the differences in environmental conditions between the populations. A blue line represents the relationship between the Mantel correlation indices and the environmental distances, and the doted lines represent the confidence intervals for this relationship. We can see that for populations that share the same environmental conditions their *Pmax* directions is more similar than expected by chance (indicated by the red line).


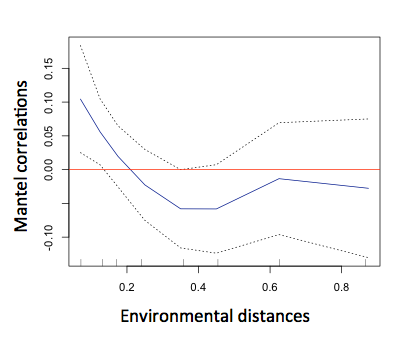


**Figure G**. **Robustness of the *G* matrix’s volume estimate to Jack-knife subsampling test**. ***G*** matrix’s volume across the species' niche as captured by the temperature gradient. Here is presented ***G***’s volume variation when removing one seed family at a time (jack-knife procedure).

|  | Biomass | Height | Shoot/Root | LDMC |
| --- | --- | --- | --- | --- |
| *M-blocks* / *S-block* effect | p-val<0.01 | p-val=0.014 | p-val<0.01 | – |
| *M-blocks* effect only | p-val<0.01 | p-val<0.01 | p-val<0.01 | p-val=0.386 |
| *S-block* effect only | p-val<0.01 | p-val<0.01 | p-val<0.01 | p-val<0.01 |

**Table B.** **Significance tests for random effects in the common garden design.** The common garden was composed of three “main blocks” (hereafter called *M-blocks*), sub-divided into ten “smaller blocks” (hereafter called *S-block*). For each trait measured at the end of the experiment, these two block effects were tested to detect whether they have a significant influence on the measurements (nested effects are indicated with “/”). The results are presented in the table above.

|  | Biomass | Height | ShootRoot | LDMC |
| --- | --- | --- | --- | --- |
| Q_ST_ [*half-sibs*] (±sd)  Q_ST_ [*full-sibs*] (±sd) | 0.074 (0.005)  0.138 (0.004) | 0.078 (0.003)  0.144 (0.008) | 0.069 (0.005)  0.130 (0.009) | 0.207 (0.030)  0.343 (0.046) |

**Table C.** **Estimation of the general Q_ST_ for the four studied traits, considering that the individuals of the same family were either half- or full-sibs**. The results robustness to the low family sample size was tested via a jack-knife procedure, removing one family per population in each population, and their deviation from the full dataset results are indicated in brackets.

|  | | **Biomass** | **Height** | **ShootRoot** | **LDMC** |
| --- | --- | --- | --- | --- | --- |
| **Fixed effects**  **(coefficient estimate)** | Temperature | 13.57 | -0.102 | 2.157 | -0.021 |
|  | Temperature^2^ | -0.247 | – | -0.057 | – |
|  | Radiation | – | – | -1.409 | -0.035 |
|  | Radiation^2^ | – | -0.013 | – | – |
|  | Temperature:Radiation | – | – | 0.052 | 0.001 |
| **Random effects**  **(standard deviation)** | *Population / Family* | 0.802 | 3.952 | 0.281 | 0.005 |
|  | *M-blocks* | 1.243 | 3.657 | 0.241 | – |
|  | *S-block* | 1.226 | 2.856 | 0.137 | 0.014 |
|  | *M-blocks* / *S-block* | 0.23 | 2.532 | 0.196 | – |
| Nb. of degrees of freedom | | 97.26 | 99.73 | 99.32 | 88.71 |
| **∆AIC** | | 13.7 (***) | 2.5 (*) | 6.8 (**) | 3.5 (*) |

F-test significance codes: 0 ‘***’ 0.001 ‘**’ 0.01 ‘*’ 0.05 ‘.’0.1

**Table D.** **Coefficients of the fixed and random effects estimated for the relationship between trait and niche gradients**. In each model only the pre-identified random effects (Table B) were included. The common garden was composed of three “main blocks” (called *M-blocks*), sub-divided into ten “smaller blocks” (called *S-block*), and their nested effects are indicated with “/”. The ∆AIC scores represent the AIC difference between the fitted model and an intercept model (significance tests are indicated with stars).

| Pop ID | He | F_IS_ | F_ST_ | Suspected of adaptation (yes/no) |
| --- | --- | --- | --- | --- |
| 7 | 0.047 | 0.109 | 0.029 | Yes |
| 8 | 0.046 | 0.393 | 0.033 | Yes |
| 23 | 0.090 | 0.517 | 0.018 | No |
| 25 | 0.067 | 0.433 | 0.015 | No |
| 27 | 0.062 | 0.571 | 0.023 | No |
| 33 | 0.042 | 0.503 | 0.026 | Yes |
| 34 | 0.053 | 0.608 | 0.036 | Yes |
| 36 | 0.044 | 0.547 | 0.018 | No |
| 37 | 0.044 | 0.351 | 0.023 | No |
| 42 | 0.041 | 0.471 | 0.024 | No |
| 50 | 0.059 | 0.635 | 0.025 | No |
| 56 | 0.064 | 0.504 | 0.024 | No |
| 58 | 0.050 | 0.590 | 0.018 | No |
| 60 | 0,038 | 0.492 | 0.021 | Yes |
| 63 | 0.036 | 0.260 | 0.034 | Yes |
| 89 | 0.041 | 0.331 | 0.025 | No |
| 97 | 0.044 | 0.389 | 0.026 | No |
| 102 | 0.041 | 0.424 | 0.050 | No |
| 105 | 0.041 | 0.439 | 0.042 | No |
| 110 | 0.049 | 0.431 | 0.024 | No |
| 112 | 0.047 | 0.272 | 0.025 | No |
| 120 | 0.053 | 0.354 | 0.027 | No |
| 138 | 0.048 | 0.386 | 0.033 | No |
| 139 | 0.056 | 0.500 | 0.027 | No |
| 151 | 0.041 | 0.258 | 0.030 | Yes |
| 191 | 0.055 | 0.697 | 0.028 | No |
| 198 | 0.048 | 0.623 | 0.025 | No |

**Table E**. **Summary table of the estimated population genetics parameters**: genetic diversity H_e_, inbreeding coefficient F_IS,_ and genetic differentiation F_ST_. The last column describes whether each population was suspected of adaptation to new environmental conditions based on the comparison of climatic niche models at regional and global scales (i.e. populations predicted inside the regional niche but out the global niche are indicated by 'yes').
